# Stopping syphilis transmission in Arctic communities through rapid diagnostic testing: The STAR study protocol

**DOI:** 10.1371/journal.pone.0273713

**Published:** 2022-09-12

**Authors:** Chelsea Caya, Mathieu Maheu-Giroux, Yiqing Xia, Bouchra Serhir, Veronique Morin, Michael Libman, Rachel Corsini, David M. Goldfarb, Tom Wong, Ameeta E. Singh, Cedric P. Yansouni

**Affiliations:** 1 Research Institute of the McGill University Health Centre, Montreal, Quebec, Canada; 2 McGill Interdisciplinary Initiative in Infection and Immunity, Montreal, Quebec, Canada; 3 Department of Epidemiology, Biostatistics and Occupational Health, School of Population and Global Health, McGill University, Montreal, Quebec, Canada; 4 Laboratoire de Santé Publique du Québec /Institut National de Santé Publique du Québec, Sainte-Anne-de-Bellevue, Quebec, Canada; 5 Nunavik Regional Board of Health and Social Services, Kuujjuaq, Quebec, Canada; 6 JD MacLean Centre for Tropical Diseases, McGill University Health Centre, Montreal, Quebec, Canada; 7 Divisions of Infectious Diseases and Medical Microbiology, McGill University Health Centre, Montreal, Quebec, Canada; 8 BC Children’s Hospital, University of British Columbia, Vancouver, British Columbia, Canada; 9 Indigenous Services Canada, Government of Canada, Ottawa, Ontario, Canada; 10 Dalla Lana School of Public Health, University of Toronto, Toronto, Ontario, Canada; 11 Department of Medicine, University of Alberta, Edmonton, Alberta, Canada; Federal University of Mato Grosso do Sul, BRAZIL

## Abstract

**Background:**

Intense transmission of syphilis has emerged in some Canadian Arctic communities despite screening and prevention efforts. The remoteness of most communities and limited diagnostic infrastructure yield long delays (≥14 days) between screening and treatment of cases. These hamper syphilis control efforts and may contribute to sustained transmission. Syphilis rapid diagnostic tests (RDTs) have been developed to make screening more accessible and to inform clinical decision-making within the same clinical encounter. These RDTs have been successfully deployed in several countries, but not yet in Canada.

**Methods and design:**

We describe the methodology of the *“Stopping Syphilis Transmission in Arctic Communities Through Rapid Diagnostic Testing”* (STAR) study, wherein the clinical and epidemiological impact of deploying a dual syphilis RDT in the context of ongoing transmission in Nunavut and Nunavik will be evaluated. In this prospective multisite field evaluation, sexually active individuals aged ≥14 years at risk for syphilis will be offered screening by an RDT at the point-of-care by non-laboratory trained registered nurses. Whole blood and serum specimens will be concurrently collected, when feasible, for rapid testing with an RDT containing both treponemal and non-treponemal components (*Chembio DPP*^*®*^ *Syphilis Screen & Confirm*) and compared to laboratory-based reference testing according to a reverse sequence algorithm. The diagnostic accuracy of the RDT, using both whole blood and centrifuged serum specimens, will be validated under real-world conditions in remote Northern settings, outside of specialized laboratories. Additionally, screening-to-treatment time, case detection rates, and the number of infectious contacts averted by using the RDT relative to reference testing will be estimated. The impact of both diagnostic approaches on syphilis transmission dynamics will also be modeled.

**Discussion:**

This study will provide much needed evidence for strengthening rapid responses to emerging syphilis outbreaks in remote Arctic regions, by supplementing traditional diagnostic strategies with an RDT to rapidly triage patients likely in need of treatment. These results will also inform the development and tailoring of future diagnostic strategies and public health responses to emerging outbreaks in the North.

## Introduction

Syphilis is a sexually transmitted infection (STI) caused by the bacterium *Treponema pallidum*, *subspecies pallidum* which, if left untreated, leads to a myriad of serious health consequences, including congenital syphilis [1,2]. In some Canadian Arctic communities, syphilis has emerged and continues unabated despite screening and prevention efforts. Prior to 2012, between 0 and 4 cases of syphilis were reported annually in the territory of Nunavut [3]. Since this initial outbreak however, syphilis has become endemic in this region with age-standardized rates of infection more than 15 times that of the rest of Canada [2,3]. Rates of infectious syphilis in Nunavut reached 234 cases per 100,000 individuals in 2017 with the highest rates reported among females (301 cases per 100,000) [4]. In the neighboring Arctic region of Nunavik in northern Quebec, an outbreak of syphilis was identified for the first time in late 2016 and incidence reached 236 cases per 100,000 in 2017 [5]. Despite intense control efforts, syphilis transmission persists in the region.

The presence of syphilis in Nunavut and Nunavik is of particular concern because these are regions with extremely high rates of other STIs such as chlamydia and gonorrhea, and have among the highest birth rates in Canada [3,5–7]. Although no cases of confirmed congenital syphilis were reported, an alarming 48 cases of infectious syphilis were identified among pregnant women in Nunavut between 2012 and 2020 [8]. Similarly in Nunavik, no confirmed congenital syphilis was identified despite 58% of new cases of syphilis in 2018 occurring among women of childbearing age [9].

Complicating disease control efforts, the remoteness of most communities in Nunavut and Nunavik hampers timely access to diagnostic testing, which is required to initiate treatment in most cases. In fact, many communities only have a nursing station without a diagnostic laboratory. As a result, the turnaround time between specimen collection and result reporting using current approaches that rely on referral to distant laboratories is typically 6 to 19 days, and is frequently longer due to additional logistical obstacles. Numerous attempts to optimize pre-analytical processes have failed to substantially reduce this interval, which is largely outside the control of the laboratory. Once positive results are reported, further delays to treatment are incurred by having to arrange a follow-up visit with newly identified cases. As outlined by a youth participant of the Consultative Committee on Sexual Health in Nunavik, “*it takes 2 weeks to get your results… that’s two Fridays*!”. The resulting long intervals between screening and subsequent treatment of infectious syphilis cases facilitates continued transmission within sexual networks and between communities. Using the contact tracing investigation records in our region, we estimate that up to half of all infectious sexual contacts may occur while screened patients are awaiting test results. Reducing the time between screening and treatment of infectious syphilis cases is therefore a key priority in these Arctic communities.

Rapid diagnostic tests (RDTs) intended to screen for syphilis have been developed to bridge this gap and have been successfully deployed in several countries, but not yet in Canada [10,11]. Syphilis RDTs are designed to be used without additional equipment and by individuals with minimal training to inform clinical decision-making within the same clinical encounter thus eliminating delays between testing and appropriate treatment and subsequent loss to follow-up. The use of a syphilis RDT as a tool for active case finding also has the potential to reduce the number of infectious contacts by accelerating treatment decisions and thereby halting any sustained undetected transmission.

Deployment of point-of-care (POC) or near-care rapid diagnostic testing would facilitate expansion of screening efforts in Arctic communities where transmission risk is identified to increase identification of cases outside of known contact networks, as no laboratory equipment or facilities are required. The current article describes our protocol that aims to evaluate the clinical and epidemiological impact of deploying a syphilis RDT in the context of ongoing transmission in Nunavut and Nunavik to inform policy for syphilis disease-control in remote Arctic settings.

## Material and methods

### Study aims

#### Specific aim 1

Evaluate the diagnostic accuracy of a syphilis RDT when used in real-world conditions in remote Arctic communities.

The accuracy of RDTs in real-world conditions can vary substantially from that observed with the same test in laboratory settings [12]. This study aim will provide reliable estimates of diagnostic accuracy in an intended-use setting and yield robust parameters for inclusion in specific aim 2. The RDT that will be used in this study is the *Chembio DPP*^*®*^ *Syphilis Screen & Confirm Assay* (Chembio Diagnostic Systems Inc., Medford, NY; Chembio RDT) [13]. Previously published evaluations of the diagnostic accuracy of the Chembio RDT indicate that when using serum, the RDT has acceptable sensitivity (≥85%) and specificity (≥94%) relative to traditional laboratory-based diagnostic approaches [14–18]. The majority of these studies however, have been conducted either in laboratory settings or by trained laboratory personnel which may bias the observed accuracy. To date, only limited data is available on its performance in the field under real-word conditions, and in high-income countries [19,20]. Furthermore, the diagnostic accuracy of venous whole blood specimens is less well characterized. This knowledge is required for deployment of the Chembio RDT in communities or settings where no centrifuge equipment is available. Therefore, participants will be asked to provide an additional single venous whole blood specimen for comparison with serum.

#### Specific aim 2

Estimate the number of cases averted when using the Chembio RDT relative to standard diagnostic algorithms and to model the impact of both diagnostic approaches on disease transmission dynamics.

Since the Chembio RDT will be used in parallel with current standard laboratory-based reference tests and diagnostic pathways, and not as a replacement, we can directly compare the impact of these two approaches. Specifically, we will quantify the added clinical value (screening coverage, case detection rates, delays between testing and appropriate treatment, and number of infectious contacts averted) of deploying this RDT as compared to traditional laboratory-based diagnostic testing. We hypothesize that the Chembio RDT with its shorter turnaround time between specimen collection and availability of results (20 minutes vs. 6–19 days with current laboratory-based algorithm), will lead to the detection of cases at earlier clinical stages, and will facilitate prompt initiation of appropriate treatment within the same clinical encounter. Early identification of asymptomatic cases through increased screening and timely treatment may interrupt the progression of syphilis and reduce of the duration of infectiousness [21].

### Study population and recruitment procedures

Sexually active individuals aged ≥14 years at risk for syphilis will be offered screening by both the RDT and standard laboratory diagnostic testing, according to local public health protocols in place.

The Chembio RDT will be implemented in (i) the sexual health clinics in two communities in Nunavik (Communities A and B) and in (ii) a nursing station in Nunavut (Community C). These clinics will provide same-day turnaround times and treatment (if required) for patients within these communities.

If new syphilis cases are detected among individuals from other communities with no previously documented syphilis transmission, or for new index cases without links to other known cases, intensified case-finding may be deployed in collaboration with local public health authorities. In such cases, dedicated and trained personnel will be deployed from Community A, B or C to perform screening in the affected communities using the RDT in parallel with a laboratory-based algorithm.

In both regions, the inclusion criteria are as follows: i) sexually active with possible syphilis (i.e., signs/symptoms) or exposure to syphilis (e.g., sexual contact of a known case), or ii) STI screening based on public health policies in place, or iii) STI screening requested by the patient, and iv) availability of adequate specimen volume to allow for testing via both diagnostic methods. All individuals screened for syphilis by both diagnostic approaches will be eligible for inclusion regardless of their test results. Individuals aged <14 years will not be eligible for inclusion in the study as they are unable to provide informed consent in accordance with local laws [22,23]. There are no other exclusion criteria.

### Ethical considerations and informed consent

The research ethics board of the Research Institute of the McGill University Health Centre has approved this study (# 2020–5834) as well as the Nunavut Research Institute (Nunavut Research License: 0300520N-M).

Authorization for use of the Chembio DPP^®^ Syphilis Screen & Confirm Assay in Canada has been obtained through Health Canada’s Special Access Program, after a robust lab-based verification of the test’s accuracy at the Quebec provincial laboratory [24], validation studies [14], and CE marking of the device. Crucially, regulatory authorization means that treatment decisions can be made on the basis of the test results. The Chembio RDT is not in itself considered investigational. Thus, when using the specimen type for which we have validated the Chembio RDT’s accuracy (i.e., serum), verbal informed consent using a script will be obtained as this laboratory implementation research is of minimal risk and follows routine clinical practices and public health policies.

There will be no financial compensation or reimbursement offered to participants. Serum specimens will be collected in all cases for standard testing for syphilis, as is currently the standard of care. For the rapid test, a positive test result indicating active syphilis infection will provide a preliminary positive result for syphilis. Participants will be treated before confirmatory testing because the Chembio RDT’s reported specificity is high, syphilis is highly infectious, and transmission is completely preventable with treatment.

Following broad community consultations, the following approach to obtaining informed consent for whole blood specimen testing was requested by the respective communities:

- In Nunavut, study participants will be read a verbal consent script listed in S1 Appendix and will then be asked to sign a full written informed consent form.
- In Nunavik, study participants will be read the verbal consent script and will not be asked to sign the written informed consent form as the local Health and Wellness Committee felt strongly that doing so would discourage syphilis testing in the community.

This study will be conducted in accordance with the latest Seoul revision of the Declaration of Helsinki (section K6b), the Medical Research Involving Human Subjects Act, the ICH Good Clinical Practice, and local regulatory requirements.

### Laboratory work

#### Specimen flow

A serum specimen will be collected from all those undergoing syphilis screening. This is the same specimen that would normally be collected for routine testing. Participants will be asked to provide an additional single venous whole blood specimen for rapid testing. This additional specimen will consist of a 10*μL* drop of venous blood collected immediately upon removal of the butterfly needle used to obtain the serum specimen for routine-care analyses.

Following initial on-site screening with the Chembio RDT, all remaining serum specimens will be stored in dedicated coolers and transported from individual villages to diagnostic laboratories according to routine local protocols. These serum specimens will then be stored at -20°C pending shipment on dry ice to their routine designated reference laboratory via airplane for standard syphilis diagnostic testing. Specimens from symptomatic and asymptomatic individuals will be tested in real-time to optimize clinical care and will be done in accordance with routine testing procedures. Specimens positive for active syphilis will be reported, as per public health law, to local public health authorities and to the relevant healthcare practitioners to guide clinical care.

In all participating communities, initial screening with the Chembio RDT will be performed during the patient clinical encounter using a venous whole blood specimen. In addition, serum specimens will be brought to the medical laboratory, or designated testing area in communities without a laboratory, and tested as soon as possible with the Chembio RDT. Results will be reported to the requesting physician and/or public health nurse and patients will be contacted (and treated if required) on the same day. Documentation and clinical follow-up of results will be integrated into routine care algorithms, under the responsibility of a designated registered nurse at participating sites.

#### Microbiological techniques

**Fig 1:**
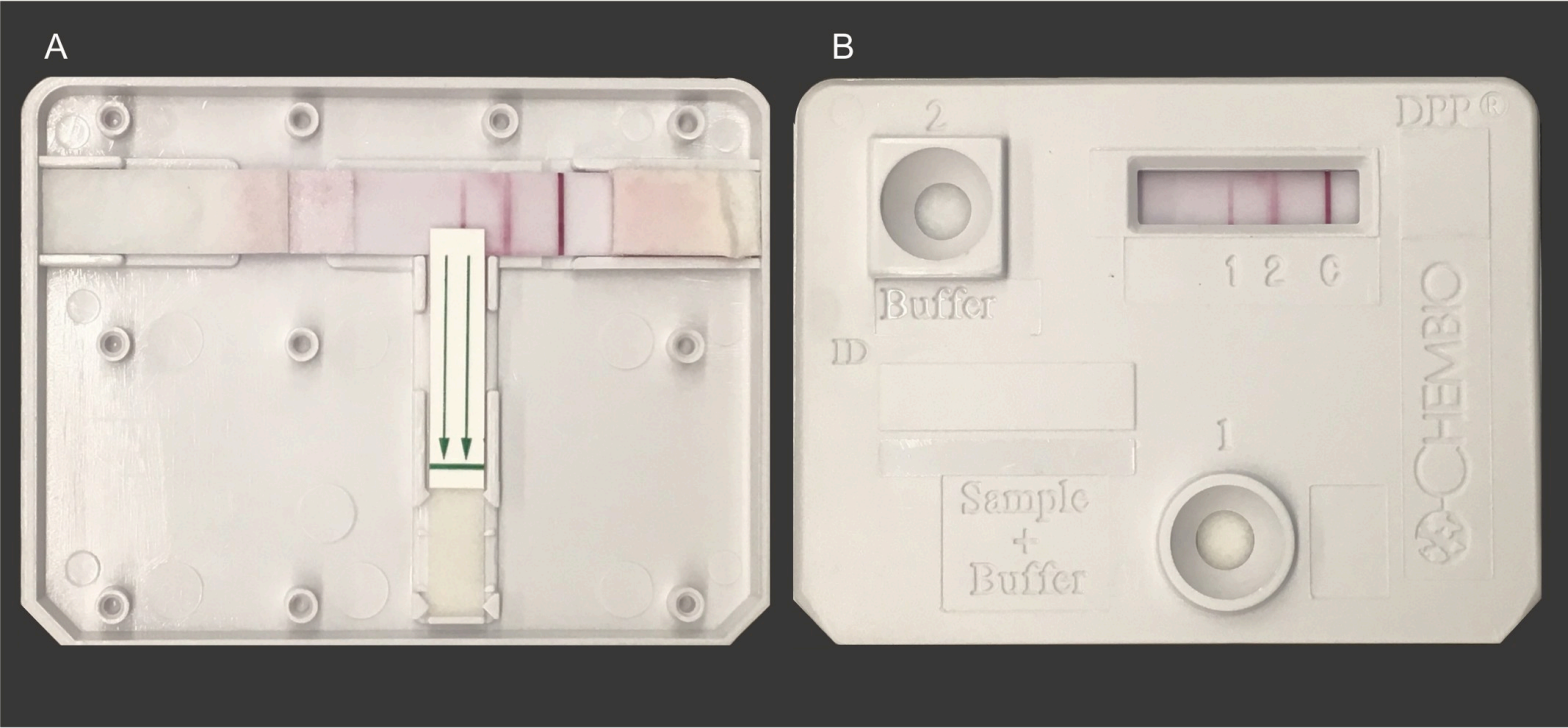
Structure of the Chembio DPP^®^ Syphilis Screen & Confirm Assay showing the locations of the antigen test lines. Panel A depicts a dissected view of the test following testing of reactive serum. Panel B depicts the complete cassette following testing of reactive serum.

Rapid testing will be performed according to the the Standard Operating Procedure detailed in the S2 Appendix. Briefly, specimens will undergo testing with the Chembio RDT according to the manufacturer’s instructions by registered nurses [13]. The Chembio DPP^®^ Syphilis Screen & Confirm Assay is designed for accurate diagnosis at the POC (Fig 1). It is a single use, rapid, immunochromatographic assay for the detection of antibodies against non-treponemal and *Treponema pallidum* antigens in human EDTA-whole blood, fingerstick blood, serum or EDTA-plasma. Results of the Chembio RDT will be interpreted by the accompanying automated digital reader to mitigate potential sources of error associated with visual interpretation of results.

In the designated provincial laboratories where confirmatory testing will be performed, the diagnosis of syphilis will be made based on the detection of antibodies against non-treponemal and treponemal antigens using routine diagnostic laboratory-based tests (Architect Syphilis TP Chemiluminescent Microparticle Immunoassay, Abbott Laboratories, Abbott Park, IL) according to a reverse sequence algorithm [25,26].

### Outcome measures

#### Specific aim 1

We will evaluate the diagnostic accuracy of the Chembio RDT when used under real-world conditions in remote Northern settings. In communities where screening is taking place, we will estimate the sensitivity and specificity of the Chembio RDT when used with serum specimens, against the reference-standard diagnostic laboratory-based reference tests that are part of the routine provincial testing algorithm.

Secondary outcomes will include (i) the positive and negative predictive values as well as the positive and negative likelihood ratios of the Chembio RDT relative to the standard laboratory-based tests; and (ii) the sensitivity and specificity of the Chembio RDT when performed using venous whole blood at the POC, as compared to the performance of the same test with serum specimens in a community-based laboratory or designated testing area.

#### Specific aim 2

The clinical and epidemiological impact of both diagnostic approaches ([rapid test + routine algorithm] vs. [routine algorithm only]) on syphilis transmission dynamics in Nunavut and Nunavik will be modeled. The model will enable us to assess the population-level consequences of identifying cases at earlier clinical stages and immediate treatment initiation, thereby resulting in shorter periods of infectiousness, with potentially important consequences on overall transmission dynamics.

### Data analysis and sample population

#### Specific aim 1

With an estimated sensitivity of 95% for the purpose of the calculation, and a precision of 5% for both syphilis components of the Chembio RDT (i.e., treponemal and non-treponemal components), we aim to recruit a minimum of 73 cases of confirmed syphilis. Given that most screened individuals will have negative results, the resulting precision around the estimate of specificity will be much higher. However, if the observed field sensitivity of the RDT falls below that observed in a pilot laboratory-based study we performed [24], we will continue recruitment until a sufficient number of confirmed cases are detected to provide an estimate of sensitivity with acceptable precision.

Descriptive analyses will be used to characterize cases overall and by region. In particular, the proportion of cases identified, the pattern of sexual contacts, and communities affected will be described for Nunavut and Nunavik. The statistical methods to compare outcomes between regions and/or between cases and non-cases may include chi-square test, Fisher’s exact test for categorical outcomes and unpaired t-test or Wilcoxon rank sum test for continuous data, as appropriate. A *P*-value of <0.05 will be considered statistically significant. The field diagnostic accuracy of the Chembio RDT will also be described (i.e., sensitivity and specificity with 95% confidence intervals). The diagnostic performance for the RDT will also be stratified by rapid plasma regain titre (non-reactive, 1:1, 1:2, 1:4, ≥1:8 dilutions) if sample size in each stratum allows.

#### Specific aim 2

Syphilis is a notifiable disease by law across Canada. As such, public health authorities in Nunavut and Nunavik closely monitor all those identified with active syphilis infection and conduct detailed patient histories and STI risk assessments [27]. When possible, sexual contacts of infectious cases are also identified as part of contact tracing and partner notification processes. As a result, we will have access to relevant denominalized individual-level data on both cases and their sexual contacts, which will be incorporated into a network analysis. Through our collaboration with the provincial laboratories in both regions, we will also have access to the results of all reference tests performed as part of the routine syphilis diagnostic algorithm (i.e., positivity and treatment outside of this study).

The clinical and epidemiological impact of both diagnostic approaches ([rapid test + routine algorithm] vs. [routine algorithm only]) on syphilis transmission dynamics in Nunavut and Nunavik will be modeled. Mathematical models of disease transmission–computer simulations of epidemics–enable investigators to capture the indirect benefits of this intervention. These indirect effects consider the longer-term public health benefits accrued at the population level by averting long chains of syphilis transmission through potentially more rapid diagnosis and treatment. Specifically, we will develop, parameterize, and calibrate a dynamic age-stratified model of syphilis transmission dynamics. The model will simulate syphilis transmission among sexually active individuals in small (<2,000 people) communities, the infection’s natural history (and infection-induced immunity against re-infection), sexual mixing characteristics, and the testing and treatment cascade [28]. The model will be calibrated within a Bayesian framework to reproduce reported sexual behaviors, the annual number of syphilis diagnoses, and the annual number of individuals screened and treated (accounting for observed diagnostic and treatment delays) in the region from 2005–2020. Once calibrated, we will compare the model predicted number of incident syphilis cases with our intervention to the incidence that would have been observed had our study not been implemented over both short- and medium-term time horizons.

Hence, the model will enable us to assess the population-level consequences of identifying cases at earlier clinical stages and immediate treatment initiation, thereby resulting in shorter periods of infectiousness, with potentially important consequences on overall transmission dynamics. Developing a mathematical model will provide us with a more in-depth understanding of the impact of deploying the syphilis RDT in terms of the number of potential cases averted relative to the current routine diagnostic algorithm. The model will additionally provide us with insights into the number of individuals treated due to false positives and those that were missed by the rapid test.

### Data handling and record keeping

This study aims to collect data on a notifiable infection. Relevant variables about cases and contacts are already collected by public health authorities. Thus, study data for analysis will be obtained from these sources. All unique identifiers (name, date of birth and provincial/territorial health care number) will be removed prior to data analysis. Anonymized data (patient demographics, dates of testing/diagnosis/treatment, diagnosis, treatment(s) prescribed, number of sexual contacts) will be collected and managed using REDCap electronic data capture tools hosted at the Research Institute of the McGill University Health Centre [29,30].

### Study status

This study is ongoing. Participant recruitment began January 16, 2020 and as of January 21, 2022, a total of 176 individuals have been recruited (144 from Nunavik and 32 from Nunavut) of which 46 new cases of syphilis have been identified. We anticipate recruitment will end in late 2022, after which data analysis and modeling will begin.

## Discussion

This project will provide much needed insight into the clinical and epidemiological impact of implementing rapid diagnostic testing in parallel with traditional laboratory-based diagnostic algorithms in response to a novel syphilis outbreak as well as in regions where syphilis has emerged/resurged in the Canadian Arctic. Our study builds upon the current evidence-base and addresses critical knowledge gaps by estimating the diagnostic accuracy and impact of the Chembio RDT on syphilis management and transmission in a population that is woefully underserved by existing literature. Accordingly, this project offers a better understanding of the exceptional challenges associated with diagnostics in the Arctic and proposes a promising solution to mitigate such difficulties.

This study will provide a strong basis in evidence for rapid responses to emerging syphilis outbreaks in remote northern regions, by supplementing traditional diagnostic strategies with an RDT to rapidly triage patients likely in need of treatment. The addition of a rapid test provides an opportunity for same-day testing and treatment in order to halt ongoing transmission to benefit individual patients and communities alike. This study will contribute the necessary evidence to inform the development and tailoring of future diagnostic strategies and public health responses to emerging outbreaks in the North as well as to improve current diagnostic responses in remote Arctic regions and beyond where syphilis is currently endemic.

## Supporting information

S1 Appendix Verbal informed consent script. (DOCX)

S2 Appendix Standard operating procedure. (DOCX)

## Abbreviations

Chembio RDT: Chembio DPP® Syphilis Screen and Confirm Assay
POC: point-of-care
RDT: rapid diagnostic test
STI: sexually transmitted infection
