# Supplementary material for: Stopping syphilis transmission in Arctic communities through rapid diagnostic testing: The STAR study protocol
Source: PLoS One. 2022 Sep 12;17(9):e0273713. doi: 10.1371/journal.pone.0273713 (PMC9467359; doi:10.1371/journal.pone.0273713)
Supplement: S1 Appendix — (DOCX) [file pone.0273713.s001.docx]

# **S1 Appendix: Verbal informed consent script**

**Study Title: Stopping syphilis transmission in Arctic communities through rapid diagnostic testing: The STAR study**

**Principal Investigators: Dr. Cedric Yansouni & Dr. Ameeta Singh**

**REB No.: XXX**

**Version/Date: XXX/XXX**

**INTRODUCTION & INVITATION**

Hello, my name is___________. I am a (nurse/community health representative) from __________ assisting with a project which aims to stopping syphilis transmission in Arctic communities. Your participation in this study is voluntary which means that you do not have to participate in this study if you do not want to.

**BACKGROUND & OBJECTIVE**

Syphilis is a sexually transmitted infection which has serious health consequences that may result in death. The infection can also be passed on to unborn children if the mother is infected.

In this study we are using a *rapid diagnostic test* which can provide results within 15-30 minutes. In addition to the rapid test, you will also be tested using the standard laboratory method.

**PROCEDURES**

During this study, a nurse or a community health representative will ask you some questions about yourself and any risks you may have for getting syphilis.

Then, a blood sample which will be used to test for syphilis will be taken from you using a needle. After 30-45 minutes, you will be told the result of the rapid test and it will be used to help decide if you need treatment for syphilis. Treatment may be given to you at the same visit.

**COMPENSATION**

Please note that you will not receive financial compensation (money) for participating in this research study. The treatment is not part of the research; only the rapid diagnostic test is.

**RISKS/DISCOMFORTS**

It is possible that the rapid test may indicate that you require treatment and that the test in the lab tells us later that you did not. In this case, it may be possible that you will receive treatment even if you are not infected with syphilis. The treatment we provide is through an injection with a common antibiotic called penicillin. It is also possible that the rapid test may indicate that you do not require treatment and that the test in the lab tells us later that you do. In this case, you will receive treatment as per routine care in the usual timeframe.

**VOLUNTARY PARTICIPATION**

Your participation in this research project is voluntary. Therefore, you may refuse to participate. You may also withdraw from the project at any time, without giving any reason, but must inform the study doctor or a member of the research team.

**CONFIDENTIALITY**

The study doctor and their team will collect and record information about you in a study file while you participate in the study. However, all of this information will be kept strictly confidential. According to public health law, syphilis is a notifiable disease and thus positive cases must be disclosed to local public health authorities.

**CONTACT INFORMATION**

If you have questions or if you have a problem you think may be related to your participation in this research study, or if you would like to withdraw, you may communicate with the study doctors or with someone on the research team at the following numbers: Dr. Yansouni: +1 (###) ###-#### or Dr. Singh: +1 (###) ###-####.

**PERMISSION TO PROCEED**

Do you have any questions or would like any additional details? *[Answer questions.]*

Do you agree to participate in this study knowing that you can withdraw at any point with no consequences to you?

*[If yes, record that participant has agreed to be part of study.]*

*[If no, thank the participant for their time.]*

[RECORD POTENTIAL PARTICIPANT’S RESPONSE]

Yes

No

_______________________________

Name of person obtaining consent

_______________________________

Signature of person obtaining consent

_______________________________

Date

_______________________________

Participant Name
