# Supplementary material for: Stopping syphilis transmission in Arctic communities through rapid diagnostic testing: The STAR study protocol
Source: PLoS One. 2022 Sep 12;17(9):e0273713. doi: 10.1371/journal.pone.0273713 (PMC9467359; doi:10.1371/journal.pone.0273713)
Supplement: S2 Appendix — (DOCX) [file pone.0273713.s002.docx]

# **S2 Appendix: Standard Operating Procedure**

| **Microbiology Laboratory** | |
| --- | --- |
| **Title: *SOP Chembio Syphilis DPP in Nunavik and Nunavut – STAR study*** | |
| **Document No: n/a** | **Version No: 1.3** |

| **Site:** Nunavik and Nunavut study communities | **Effective date:** 2019-09-01 |
| --- | --- |
| **Manual:** STAR Study Manual | **Status:** active |

TABLE OF CONTENTS

[1.0 PURPOSE AND PRINCIPLE 4](#_Toc93321312)

[2.0 CLINICAL SPECIMEN 4](#_Toc93321313)

[3.0 RECEPTION 4](#_Toc93321314)

[4.0 EQUIPMENT & MATERIAL 6](#_Toc93321315)

[5.0 ROLES & RESPONSIBILITIES: 7](#_Toc93321316)

[6.0 TEST PROCEDURE 8](#_Toc93321317)

[7.0 INTERPRETATION OF RESULTS 13](#_Toc93321318)

[8.0 PERFORMANCE PARAMETERS 15](#_Toc93321319)

[9.0 QUALITY ASSURANCE 16](#_Toc93321320)

[10.0 RESULTS REPORTING 17](#_Toc93321321)

[11.0 LIMITATIONS OF THE PROCEDURE 18](#_Toc93321322)

[12.0 REFERENCES 18](#_Toc93321323)

[13.0 APPENDICES 18](#_Toc93321324)

[APPENDIX 1: Quality control record for DPP Syphilis Screen & Confirm Assay 19](#_Toc93321325)

[APPENDIX 2: Results table for clinical specimens - DPP Syphilis Screen & Confirm Assay 20](#_Toc93321326)

Document Historical Record

| Version | Effective date: | Description of revisions: | Approved by: |
| --- | --- | --- | --- |
| **1.1** | 2019/08/30 | Note. Consensus to use 10 µl of serum instead of 5 ul was made by C.Yansouni and B.Serhir following in-laboratory pilot validation | C.Yansouni |
| 1.2 | 2020/08/17 | Note. Added details on how to prepare control sera for quality control testing -section 6.1 | C.Yansouni |
| 1.3 | 2021/04/12 | Updated result reporting documents  (appendix 1 & 2) and section 8.2 | C.Yansouni |
|  |  | <List major changes or revisions requiring new version.  Changes can also be highlighted. > |  |

# PURPOSE AND PRINCIPLE

The purpose of this standard operating procedure is to outline the procedures required to test for syphilis using the Chembio DPP^®^ Syphilis Screen & Confirm Assay (Chembio Diagnostic Systems Inc., Medford, NY) in Nunavik and Nunavut. Syphilis is a chronic, multistage sexually transmitted infection caused by the spirochete Treponema pallidum. Clinical manifestations of syphilis include both congenital syphilis and acquired syphilis. Acquired syphilis can be divided into three stages which may ultimately lead to damage of the cardiovascular and/or the nervous system if left undetected and untreated. Syphilis transmission is ongoing in Nunavik and Nunavut. These regions have among the highest rates of sexually transmitted infections in Canada and timely access to diagnostics is difficult due to the remoteness of most communities. Many of these communities have a nursing station without a lab capable of screening and testing for syphilis in a timely fashion. Typical turnaround times for test results are sometimes greater than 2 weeks, allowing for ongoing transmission within and between communities. **It is vital to curtail further transmission of this serious disease by every means necessary**. The Chembio DPP^®^ Syphilis Screen & Confirm Assay is a single-use immunochromatographic rapid screening test for the simultaneous detection of antibodies against non-treponemal and *Treponema pallidum* antigens in human fingerstick whole blood, venous whole blood, serum or plasma specimens. The DPP^®^ Syphilis Screen & Confirm Assay is intended for use as a point-of-care (POC) test to aid in the screening and/or confirmation of infection with syphilis. Authorization for use has been granted by Health Canada under the Special Access Program.

# CLINICAL SPECIMEN

The primary specimen type used in the STAR study will be:

- Serum (centrifuged yellow-top serum separator tube – 1,300g x 15 minutes)
  - Serum specimens can be tested upon collection and centrifugation. If testing is not performed immediately, they should be stored at 2 to 8ºC as soon as possible for up to 7 days. If testing of serum specimens is not performed within 7 days, then specimens should be frozen as soon as possible at -20ºC.
- Plasma may be substituted for serum if serum cannot be obtained (clotted uncentrifuged yellow-top gel tube)
- During the study, a subset of patients to be defined may provide ADDITIONAL whole blood specimens for comparison with serum. In these cases, the following specimens may be used in addition to serum:
  - Fingerstick whole blood (test performed at time of collection)

**or**

- - Venous whole blood (lavender tube, test performed within 15 minutes of collection)

# RECEPTION

- 1. **Transport:**
- Specimens should be transported to the site of testing in a dedicated cooler using standard lab safety practices and should be packed in compliance with regulations covering the transportation of etiologic agents. Venous whole blood, serum and plasma specimens should be shipped refrigerated with cold packs or wet ice.
- Each labelled specimen should be packaged in a leak-proof biohazard bag prior to placing in a transport cooler, according to local protocols.
  1. **Handling**
- Handle the specimens and materials in contact with specimens as if capable of transmitting infection.
- Do not eat, drink or smoke in the area where specimens and kit reagents are handled. Avoid any contact between hands, eyes or mouth during specimen collection and testing.
- Wear protective clothing such as laboratory coats, disposable gloves and eye protection when handling patient specimens.
- Dispose of all specimens and materials used in the test procedure in a biohazard waste container. Lancets should be placed in a puncture-resistant container prior to disposal. The recommended method of disposal of biohazard waste is autoclaving for a minimum of 1 hour at 121ºC. Disposable materials may be incinerated. Liquid wastes may be mixed with appropriate chemical disinfectants. A freshly prepared solution of 10% bleach (0.5% solution of sodium hypochlorite) is recommended. Allow 60 minutes for effective decontamination.
- Do not autoclave solutions that contain bleach.
- Use 10% bleach or other appropriate disinfectants to wipe all spills. The bleach solution should be made fresh each day.
- For in vitro diagnostic use, the test should be performed at 18 to 30°C.
  1. **Acceptance Criteria**

Specimens must be received in proper packaging and have adequate labelling. For further details, please refer to the acceptance criteria listed in the standard operating procedure for your region.

- 1. **Rejection Criteria**
- Unlabeled specimens should be rejected.
- Specimens kept at room temperature for more than 24 hours prior to testing

should be identified with a comment.

# EQUIPMENT & MATERIAL

- 1. **Equipment (Required)**
- DPP® Syphilis Screen & Confirm Individually Pouched Test Devices
- Disposable Microsafe® Tubes
- DPP® Syphilis Running Buffer Bottle (6mL) – Red cap
- Product Insert
- 1 Chembio DPP® Micro Reader (includes 3 batteries) (REF: 70-1001-0)
- 15 Additional CR2032 3V Lithium Batteries: CR2032 (3V/230 mAh)
- 1 Holder for use with DPP® Test Device
- 1 USB Power Adapter (5V/1000 mA)
- 1 Certificate of Analysis
- 1 RFID Card for use with DPP® Syphilis Screen & Confirm Assay (REF: 70-1055-0)
  1. **Material (Required)**
- Clock, watch or other timing device
- Pipettor capable of delivering 5μL of specimen for serum or plasma specimens
- Pipettor capable of delivering 10μL of specimen for venous specimens
- Sterile gauze (for fingerstick specimens only)
- Disposable gloves
- Antiseptic wipes
- Biohazard disposal containers
- Sterile Safety Lancet (for fingerstick specimens only)
- Collection devices for specimens other than fingerstick

# ROLES & RESPONSIBILITIES:

**Nunavik communities:**

| Patients to be screened are identified according to which policy: | According to the local public health policies already in place |
| --- | --- |
| Patients informed consent to obtained by: | Local public health nurse |
| Specimens will be collected by: | Local public health nurse |
| Specimens will be labelled by: | Local public health nurse |
| Specimen will be processed (centrifugation) by: | Local public health nurse |
| Testing will be performed by:  At [location]  Within [turnaround time] | Local public health nurse |
|  | The clinical laboratories in Communities A and B |
|  | Same day |
| Results will be documented by: | Local public health nurse |
| Patients will be contacted within [time]  and if indicated, offered treatment by: | Same day as testing |
|  | Local public health nurse according to local public health protocols |

**Nunavut community:**

| Patients to be screened are identified according to: | According to the local public health policies already in place |
| --- | --- |
| Patients informed consent to obtained by: | Local public health nurse |
| Specimens will be collected by: | Local public health nurse |
| Specimens will be labelled by: | Local public health nurse |
| Specimen will be processed (centrifugation) by: | Local public health nurse |
| Testing will be performed by:  At [location]  Within [turnaround time] | Local public health nurse |
|  | Local nursing station in Community C |
|  | Same day |
| Results will be documented by: | Local public health nurse |
| Patients will be contacted within [time]  and if indicated, offered treatment by: | Same day as testing |
|  | Local public health nurse according to local public health protocols |

# TEST PROCEDURE

- 1. **Specimen Collection**
     - For Serum or Venous Blood Specimens, use an accurate laboratory pipette to obtain the required amount of specimen:
- Serum or plasma – 10 µL
- Whole blood – 10µL
  - - For fingerstick whole blood, prick the finger and wipe away the first drop. Collect the second drop with a Microsafe® Tube holding it in a horizontal position as shown. Touch the tip of the tube to the blood specimen. Capillary action will draw the specimen to the black fill line and stop.


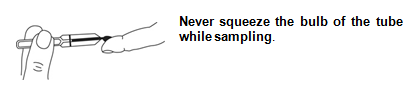


If the specimen and/or kit components have been refrigerated, remove them from the refrigerator and allow them to come to a temperature of 18 to 30ºC (64 to 86ºF) prior to testing.

*If using frozen serum samples intended for quality control assessment, please do the following*

- Remove one positive and one negative control serum sample for quality control testing and let them thaw at room temperature for at least 15 minutes.
- Once thawed, centrifuge/vortex both control serum samples to ensure the samples are well mixed (10,000g x 10 min). Proceed as usual.
  1. **Transfer of the Specimen to the Testing Device**

Transfer the required amount of serum, plasma or whole blood into the center of the round SPECIMEN + BUFFER Well 1 of the device.

For fingerstick blood, transfer the blood from the Microsafe Tube into the center of the round SPECIMEN + BUFFER Well 1 of the device as shown below.

NOTE: the diagrams below refers to fingerstick blood ONLY

NOTE: For each specimen, be sure to ALWAYS use a NEW pipette tip to avoid contamination.

**
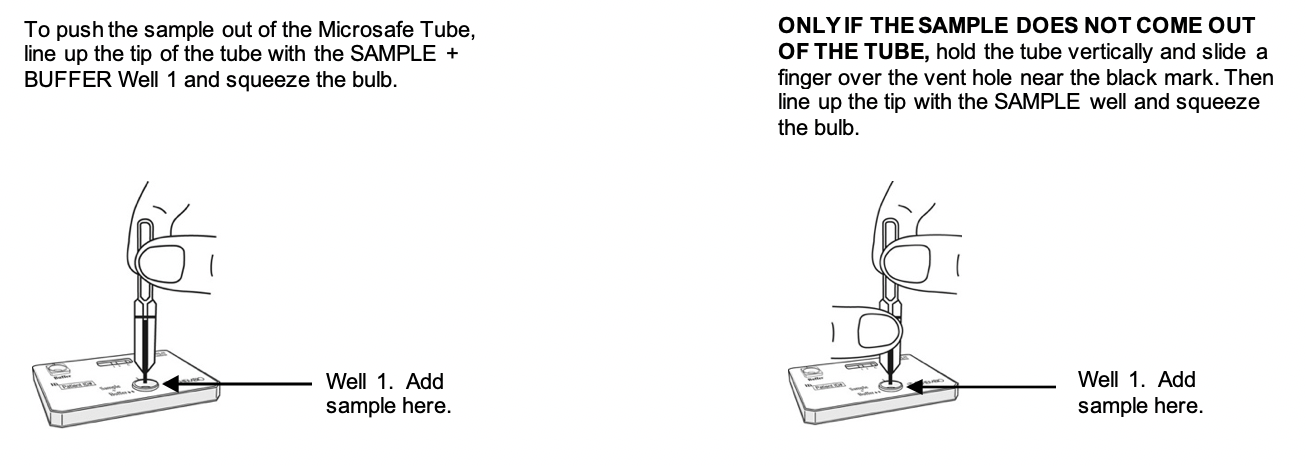
**

- 1. **Transfer of the Running Buffer to the Testing Device**

Invert the Running Buffer bottle (red cap) and hold it vertically (not at an angle) over SPECIMEN + BUFFER Well 1. Add 2 drops of Running Buffer (~50µL) into the center of the round SPECIMEN + BUFFER Well 1 (see below).

NOTE: When removing the cap from the buffer bottle, take care to remove only the red portion of the cap. Do not unscrew the white part from the bottle.

**
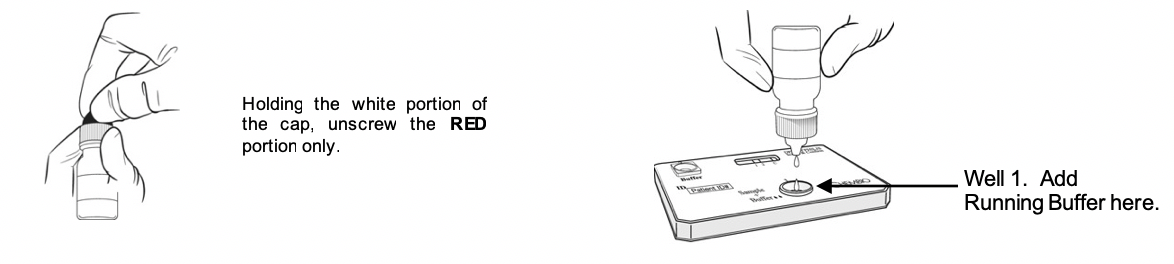
**

**Wait 5 minutes.** The blue and green colored lines should have disappeared from the rectangular window. If not, discard the test device and repeat the procedure with a new test device.

Invert the Running Buffer bottle (red cap) and hold it vertically (not at an angle) over BUFFER Well 2. Slowly add 5 drops (~135µL) of Running Buffer to BUFFER Well 2 (see below). Start timing device for **15 minutes**.


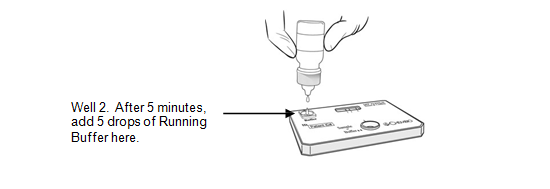


- 1. **Running Test without DPP MICRO READER**

[NOTE: DPP^®^ MICRO READER should always be used if available. This procedure is included in case of missing or malfunctioning reader] After the addition of Running Buffer into BUFFER Well 2, read the test result after waiting 15 minutes. In some cases, a test line may appear in less than 15 minutes. However, 15 minutes are needed to report a non-reactive result. Do not read results after more than 15 minutes from start of test (Start of test = First addition of Running Buffer added to Well 2).

NOTE: Discard the used test device and any other test materials into a biohazard waste container.

- 1. **Running test with DPP^®^ MICRO READER**

Check to make sure that the window at the bottom of the reader is clean of finger marks and dust or lint before using the reader. The reader and holder assembly must be on top of the test device when reading the device for results to be valid.

1. Connect the DPP^®^ MICRO READER to the supplied holder as shown below. Insert the base of the reader so that the “slanted” edge meets the corresponding “slanted” corner in the cartridge holder. The reader should lay flat in the holder and the button and battery compartment should face the specimen well, and the user.


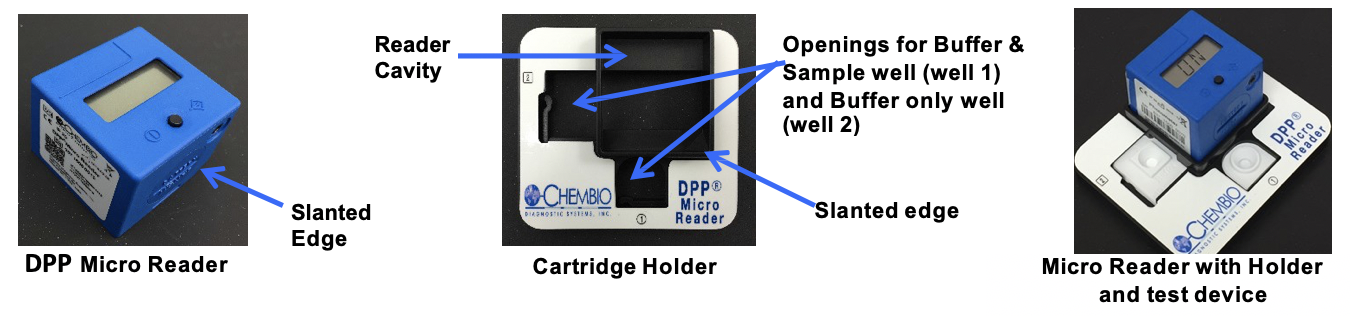


1. At the time indicated for reading the test results:

Place the reader and holder assembly on top of the test device cassette and press the center button briefly (less than one second).

“ON” should appear in the display window.


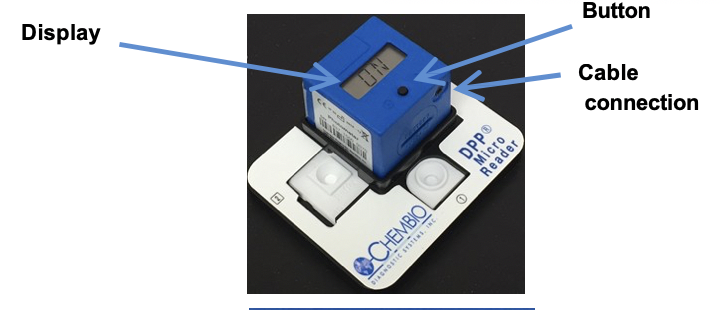


1. Press the button again and the display will then call for the RFID card


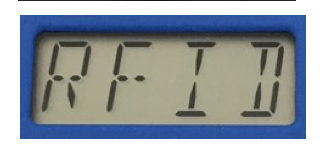


1. Place the RFID card on top of the reader to allow the reader to obtain assay information and then remove it after the alarm indicates that the card was successfully read.


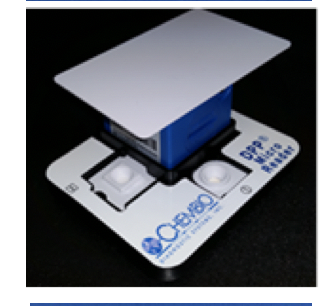


1. Upon removing the RFID card, the display will then read “TEST”


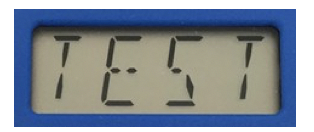


1. Press the button one more time (<1 second) and the reader will display “RUN”


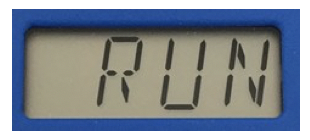


1. After a few seconds and audible alarm occurs and the results of the DPP test will scroll across the display. Write down the results; refer to the “Interpretation of Results” section.

NOTE: Results will be displayed for approximately 50 seconds before the reader shuts off.

# INTERPRETATION OF RESULTS

**Interpretation of Results with DPP^®^ MICRO READER**

| **Result** | **Interpretation** | **What you will see on reader** |
| --- | --- | --- |
| **NONREACTIVE FOR BOTH**  **TREPONEMAL**  **&**  **NON-TREPONEMAL** | The test result is interpreted as Preliminary NEGATIVE for Treponemal and non-treponemal antibodies. | ***TRP < 9 / NR NTP < 9 / NR***  *Will scroll across display screen* |
| **REACTIVE for BOTH:**  **TREPONEMAL**  **&**  **NON-TREPONEMAL** | The test result is interpreted as Preliminary POSITIVE for Treponemal and non-Treponemal antibodies. | ***TRP ≥ 9 / R NTP ≥ 9 / R***  *Will scroll across display screen* |
| **REACTIVE:**  **TREPONEMAL**  **&**  **NONREACTIVE:**  **NON-TREPONEMAL** | The test result is interpreted as Preliminary POSITIVE for Treponemal antibodies. | ***TRP ≥ 9 / R***  ***NTP < 9 / NR***  *Will scroll across display screen* |
| **NONREACTIVE:**  **TREPONEMAL**  **&**  **REACTIVE:**  **NON-TREPONEMAL** | The test result is interpreted as Preliminary NEGATIVE for Treponemal antibodies. | ***TRP < 9 / NR***  ***NTP ≥ 9 / R***  *Will scroll across display screen* |
| **INVALID** | The test is INVALID. An INVALID test cannot be interpreted.  INVALID test should be repeated once with a new device.  If the same result occurs, then the result is reported as “INVALID result, specimen sent to reference lab” | ***INV***  *Will scroll across display screen* |

**Visual Interpretation of Test Results (without DPP^®^ MICRO READER)**

NB: Visual interpretation should *ONLY* be used when DPP^®^ MICRO READER is NOT available.

| **NONREACTIVE FOR BOTH**  **TREPONEMAL**  **&**  **NON-TREPONEMAL** | One pink/purple line in the CONTROL (C) area with no line in the TEST (1) or TEST (2) areas indicates a nonreactive result. A nonreactive result at 15 minutes from the addition of Running Buffer to Well 2 indicates that there are no detectable antibodies to both Treponemal and non-Treponemal antigens. The test result is interpreted as Preliminary NEGATIVE for Treponemal and non-Treponemal antibodies. | 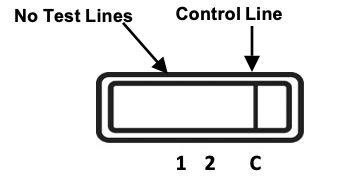 |
| --- | --- | --- |
| **REACTIVE for BOTH:**  **TREPONEMAL**  **&**  **NON-TREPONEMAL** | Three pink/purple lines, one in the TEST (1) area, one in the TEST (2) area, and one in the CONTROL (C) area indicate a reactive result for Treponemal and non-Treponemal antibodies. The lines in the TEST areas may look different from the line in the CONTROL (C) area and the two TEST lines may look different from each other. Intensities of the TEST (1), TEST (2) and CONTROL (C) lines may vary. Test results with visible lines in TEST (1), TEST (2) and CONTROL (C) areas, regardless of intensity, are considered REACTIVE for both Treponemal and non-Treponemal antibodies. The test result is interpreted as Preliminary POSITIVE for Treponemal and non-Treponemal antibodies. | 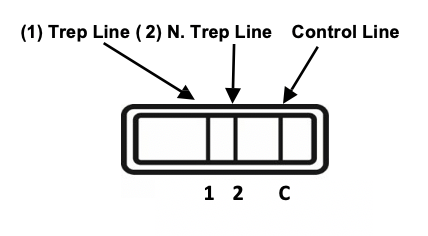 |
| **REACTIVE:**  **TREPONEMAL**  **&**  **NONREACTIVE:**  **NON-TREPONEMAL** | Two pink/purple lines, one in the TEST (1) area and one in the CONTROL (C) area indicate a reactive result for Treponemal antibodies. The line in the TEST area may look different from the line in the CONTROL (C) area. Intensities of the TEST (1) and CONTROL (C) lines may vary. Test results with visible lines in TEST (1) and CONTROL (C) areas, regardless of intensity, are considered REACTIVE for Treponemal antibodies. The test result is interpreted as Preliminary POSITIVE for Treponemal antibodies. | 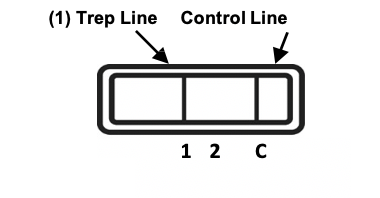 |
| **NONREACTIVE:**  **TREPONEMAL**  **&**  **REACTIVE:**  **NON-TREPONEMAL** | Two pink/purple lines, one in the TEST (2) area and one in the CONTROL (C) area indicate a reactive result for non-Treponemal antibodies. The line in the TEST area may look different from the line in the CONTROL (C) area. Intensities of the TEST (2) and CONTROL (C) lines may vary. Test results with visible lines in TEST (2) and CONTROL (C) areas, regardless of intensity, are considered REACTIVE for non-Treponemal antibodies. The test result is interpreted as Preliminary NEGATIVE for Treponemal antibodies. | 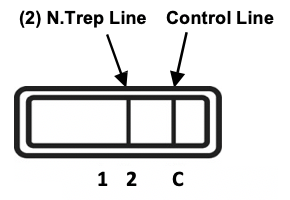 |
| **INVALID** | A pink/purple line should always appear in the CONTROL (C) area whether or not one or two lines appear in the TEST areas. If there is no distinct pink/purple line visible in the CONTROL (C) area, then the test is INVALID. An INVALID test cannot be interpreted. It is recommended that the INVALID test be repeated with a new device. | ***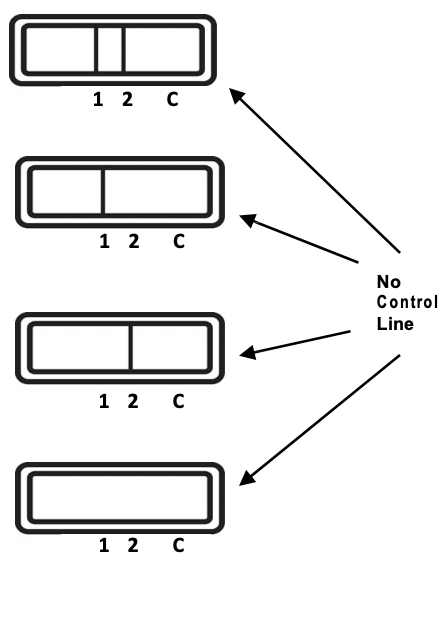*** |

# PERFORMANCE PARAMETERS

- 1. **Calibration:** None
  2. **Quality Control**
     - Each testing site will receive a quality control (QC) panel of known positive and negative controls.
     - Positive and negative control sera should be used to verify the performance of tests in each of the following situations:
       - Every time a new shipment is received
       - Every time the lot number changes
       - Once per 2-weeks if no more recent QCs have been performed
     - Results of QC are recorded in **APPENDIX 1**, stored on-site, and PDF copies sent to the study coordinator at regular intervals (e.g., once per month).
- Below are examples of how to interpret results for quality control purposes:

| Control specimen | RDT  Treponemal result | RDT  Non-Treponemal Result | Interpretation |
| --- | --- | --- | --- |
| Positive | Reactive (+) | Reactive (+) | PASS |
| Positive | Reactive (+) | Non-Reactive (-) | FAIL |
| Positive | Non-Reactive (-) | Reactive (+) | FAIL |
| Negative | Non-Reactive (-) | Non-Reactive (-) | PASS |
| Negative | Reactive (+) | Non-Reactive (-) | FAIL |
| Negative | Non-Reactive (-) | Reactive (+) | FAIL |

- In the event that QC fails, please do the following:
- Review your QC records to see if the same control specimen was used previously and compare the results. If testing was done previously with the same control specimen and it passed QC, the specimen may no longer be viable/usable.
- Repeat testing using the same lot of rapid test with a different control specimen to help further assess the cause of the QC failure. If the new control specimen passes QC, then the failure was likely caused by the previous control specimen which should be discarded.
- Report any QC failures to the study coordinator via email as soon as possible.
  1. **Storage Requirements**
     - The DPP^®^ Syphilis Screen & Confirm Assay devices should be stored in unopened pouches at 2 to 30°C (36 to 86°F). Do not freeze.
     - Do not open pouch until you are ready to perform a test.
     - When stored as indicated, test devices are stable until the expiration date marked on the pouch.
     - Running Buffer should be stored at 2 to 30°C (36 to 86°F) in its original bottle. Do not use beyond the indicated expiration date.
     - Venous whole blood, serum and plasma specimens may be tested immediately after collection. If specimens are not tested immediately, refrigerate them at 2 to 8°C (36 to 46°F) following collection. These specimens should be tested within 1 day of collection, otherwise serum or plasma specimens should be frozen at -20°C (-4°F) or colder.
     - Allow refrigerated specimens to reach room temperature and mix gently before testing.
     - DO NOT FREEZE WHOLE BLOOD!

# QUALITY ASSURANCE

9.1 The Chembio DPP^®^ Syphilis Screen & Confirm Assay is always performed in parallel with reference laboratory techniques according to STAR study and current public health protocols. As such, each specimen has its own external quality assurance panel, and the results of referral laboratory testing will define the reference standard.

# RESULTS REPORTING

- 1. **Critical Results**
- For results of the Chembio DPP^®^ Syphilis Screen & Confirm Assay interpreted as “preliminary POSITIVE”, follow current public health procedures for syphilis testing.
  1. **Preliminary Report**
- RECORDING: Record results from each column in APPENDIX 2
- REPORTING: For tests performed in communities without a laboratory, report Chembio DPP^®^ Syphilis Screen & Confirm Assay as follows:
- Negative results:

“*PRELIMINARY NEGATIVE result using rapid diagnostic test*”

- preliminary POSITIVE results:

*“PRELIMINARY POSITIVE result:*

*Rapid diagnostic test Treponemal result: Reactive*

*Rapid diagnostic test Non-Treponemal result: [*specify *Reactive* or *Non-reactive]”*

- Invalid results:

“*The result of the rapid diagnostic test is invalid*”

- For all reports, add the comment “*Rapid diagnostic test (Chembio DPP Syphilis Screen & Confirm Assay) performed in [name community]; results of routine syphilis tests to follow*”
  1. **Final Report**
- In addition to above result, **ADD** results of standard testing according to current protocols

# LIMITATIONS OF THE PROCEDURE

- The Chembio DPP^®^ Syphilis Screen & Confirm Assay must be used with human capillary (fingerstick) or venous whole blood, serum, and plasma only. Using other types of specimens or testing of venipuncture whole blood specimens collected using a tube containing an anticoagulant other than citrate, heparin or EDTA may not yield accurate results. For serum specimens, collect blood without anticoagulant.
- The Chembio DPP^®^ Syphilis Screen & Confirm Assay must be used in accordance with the instructions in this product insert to obtain accurate results.
- Test results must be read at 15 minutes after the addition of Running Buffer to the BUFFER Well 2 (Step 5).
- Ensure finger is completely dry before performing fingerstick.
- Read results in a well-lit area.
- A reactive result using the Chembio DPP^®^ Syphilis Screen & Confirm Assay suggests the presence of antibodies to syphilis *Treponema pallidum* and/or non-treponemal antigens.
- For a reactive result, the intensity of the test line does not necessarily correlate with the titer of antibody in the specimen.
- A non-reactive result does not preclude the possibility of exposure to syphilis or infection with syphilis. An antibody response to recent exposure may take several months to reach detectable levels.

# REFERENCES

Bennett, J. E., Dolin, R., & Blaser, M. J. (2017). *Mandell, Douglas, and Bennett’s infectious disease essentials.*

Chembio. *Next Generation DPP^TM^ DPP***®** *Syphilis Screen & Confirm Assay.* Medford NY.

# APPENDICES

Appendix 1: Quality control record for DPP Syphilis Screen & Confirm Assay

Appendix 2: Results table for clinical specimens - DPP Syphilis Screen & Confirm Assay

## **APPENDIX 1: Quality control record for DPP Syphilis Screen & Confirm Assay**

|  | |  | |  | Chembio RDT Results | | | |  |  |  |  |
| --- | --- | --- | --- | --- | --- | --- | --- | --- | --- | --- | --- | --- |
|  | |  | |  | **Treponemal Results** | | **Non-Treponemal** | |  |  |  |  |
| Control specimen ID  (e.g. Pos ctrl #1) | | Date of QC Testing  (AAAAMMJJ)  (YYYYMMDD) | | Lot # | Expiry/  Peremption  Date  (AAAAMMJJ)  (YYYYMMDD) | Quantifi-  cation* | Interpre-  tation  (R/NR) | Quantifi-  cation* | Interpre-  tation  (R/NR) | Interpre-  tation  (PASS / FAIL) | Signature of person who performed QC | Comments |
|  | |  | |  |  |  |  |  |  |  |  |  |
|  | |  | |  |  |  |  |  |  |  |  |  |
|  | |  | |  |  |  |  |  |  |  |  |  |
|  | |  | |  |  |  |  |  |  |  |  |  |
|  | |  | |  |  |  |  |  |  |  |  |  |
|  | |  | |  |  |  |  |  |  |  |  |  |
|  | |  | |  |  |  |  |  |  |  |  |  |
|  | |  | |  |  |  |  |  |  |  |  |  |
|  | |  | |  |  |  |  |  |  |  |  |  |
|  | |  | |  |  |  |  |  |  |  |  |  |
|  | |  | |  |  |  |  |  |  |  |  |  |
|  | |  | |  |  |  |  |  |  |  |  |  |

*Only enter a quantification of results that have been obtained using the DPP^®^ Micro Reader.

| **WHEN TO PERFORM QC TESTING:** | **OTHER PARAMETERS TO CONTROL** |
| --- | --- |
| - - - - Every time a new shipment is received       - Every time the lot number changes       - Once per 2-weeks if no more recent QCs have been performed       - STORE THIS SHEET AT SITE OF TESTING       - Start a new sheet if the SPECIMEN # used for controls are changed | - Devices should be stored in unopened pouches at 2 to 30°C. Do not freeze. - Control specimens should be stored at -20°C in the rear of the freezer - Do not open pouch until you are ready to perform a test. - Running Buffer should be stored at 2 to 30°C (36 to 86°F) in its original bottle. Do not use beyond the indicated expiration date. |

## **APPENDIX 2: Results table for clinical specimens - DPP Syphilis Screen & Confirm Assay**

LOT Number: _____________

|  |  |  |  |  | **Visual Results** | | **DPP^®^ Micro Reader Results** | | | | |  |
| --- | --- | --- | --- | --- | --- | --- | --- | --- | --- | --- | --- | --- |
|  |  |  |  |  | **TRP** | **Non-TRP** | **Treponemal** | | **Non-Treponemal** | | **Interpretation** |  |
| Patient  ID | Specimen  ID | Specimen type | Date de prélèvement – collection date  (AAAAMMJJ)  (YYYYMMDD) | Date de l’analyse – testing date  (AAAAMMJJ)  (YYYYMMDD) | Interpret-ation (R/NR) | Interpret-ation (R/NR) | Quanti-  fication* | Interpret-ation (R/NR) | Quanti-  fication* | Interpret-ation (R/NR) | Choose one:   - Preliminary NEGATIVE, - Preliminary POSTIVE, - INVALID, - TEST CANCELLED | Comments |
|  |  |  |  |  |  |  |  |  |  |  |  |  |
|  |  |  |  |  |  |  |  |  |  |  |  |  |
|  |  |  |  |  |  |  |  |  |  |  |  |  |
|  |  |  |  |  |  |  |  |  |  |  |  |  |
|  |  |  |  |  |  |  |  |  |  |  |  |  |
|  |  |  |  |  |  |  |  |  |  |  |  |  |
|  |  |  |  |  |  |  |  |  |  |  |  |  |
|  |  |  |  |  |  |  |  |  |  |  |  |  |
|  |  |  |  |  |  |  |  |  |  |  |  |  |
|  |  |  |  |  |  |  |  |  |  |  |  |  |
|  |  |  |  |  |  |  |  |  |  |  |  |  |
|  |  |  |  |  |  |  |  |  |  |  |  |  |
|  |  |  |  |  |  |  |  |  |  |  |  |  |
|  |  |  |  |  |  |  |  |  |  |  |  |  |
|  |  |  |  |  |  |  |  |  |  |  |  |  |

For visual results, the presence of any line regardless of the strength/density of the line, is considered reactive (R).

TRP: Treponemal results; Non-TRP: non-Treponemal results.

*Only enter a quantification of results that have been obtained using the DPP^®^ Micro Reader.
